# Quantifying climate sensitivity and climate-driven change in North American amphibian communities

**DOI:** 10.1038/s41467-018-06157-6

**Published:** 2018-09-25

**Authors:** David A. W. Miller, Evan H. Campbell Grant, Erin Muths, Staci M. Amburgey, Michael J. Adams, Maxwell B. Joseph, J. Hardin Waddle, Pieter T. J. Johnson, Maureen E. Ryan, Benedikt R. Schmidt, Daniel L. Calhoun, Courtney L. Davis, Robert N. Fisher, David M. Green, Blake R. Hossack, Tracy A. G. Rittenhouse, Susan C. Walls, Larissa L. Bailey, Sam S. Cruickshank, Gary M. Fellers, Thomas A. Gorman, Carola A. Haas, Ward Hughson, David S. Pilliod, Steven J. Price, Andrew M. Ray, Walt Sadinski, Daniel Saenz, William J. Barichivich, Adrianne Brand, Cheryl S. Brehme, Rosi Dagit, Katy S. Delaney, Brad M. Glorioso, Lee B. Kats, Patrick M. Kleeman, Christopher A. Pearl, Carlton J. Rochester, Seth P. D. Riley, Mark Roth, Brent H. Sigafus

**Affiliations:** 1 Department of Ecosystem Science and Management, Pennsylvania State University, University Park, PA 16802 USA; 2 U.S. Geological Survey, Patuxent Wildlife Research Center, SO Conte Anadromous Fish Lab, 1 Migratory Way, Turners Falls, MA 01376 USA; 3 U.S. Geological Survey, Fort Collins Science Center, Fort Collins, CO 80523 USA; 4 Intercollege Graduate Ecology Program, Pennsylvania State University, University Park, PA 16802 USA; 5 U.S. Geological Survey, Forest and Rangeland Ecosystem Science Center, Corvallis, OR 97331 USA; 6 Ecology and Evolutionary Biology Department, University of Colorado, Boulder, Boulder, CO 80309 USA; 7 U.S. Geological Survey, Wetland and Aquatic Research Center, Lafayette, LA 70506 USA; 8 School of Environment and Forest Sciences, University of Washington, Seattle, WA 98195 USA; 9 Conservation Science Partners, Seattle, WA 98102 USA; 10 Institute of Evolutionary Biology and Environmental Studies, University of Zurich, Zurich, 8057 Switzerland; 11 Info Fauna Karch, 2000 Neuchâtel, Switzerland; 12 U.S. Geological Survey, South Atlantic Water Science Center, Norcross, GA 30093 USA; 13 U.S. Geological Survey, Western Ecological Research Center, San Diego, CA 92101 USA; 14 Redpath Museum, McGill University, Montreal, QC H3A 0C4 Canada; 15 U.S. Geological Survey, Northern Rocky Mountain Science Center, Aldo Leopold Wilderness Research Institute, Missoula, MT 59801 USA; 16 Department of Natural Resources and the Environment, University of Connecticut, Storrs, CT 06269 USA; 17 U.S. Geological Survey, Wetland and Aquatic Research Center, Gainesville, FL 32653 USA; 18 Department of Fish, Wildlife and Conservation Biology, Colorado State University, Fort Collins, CO 80523 USA; 19 U.S. Geological Survey, Western Ecological Research Center, Point Reyes Station, CA 94956 USA; 20 Department of Fish and Wildlife Conservation, Virginia Tech, Blacksburg, VA 24061 USA; 21 Parks Canada, Jasper, Alberta T0E 1E0 Canada; 22 U.S. Geological Survey, Forest and Rangeland Ecosystem Science Center, Boise, ID 83706 USA; 23 Department of Forestry and Natural Resources, University of Kentucky, Lexington, KY 40506 USA; 24 Greater Yellowstone Network, National Park Service, Bozeman, MT 59715 USA; 25 U.S. Geological Survey, Upper Midwest Environmental Sciences Center, La Crosse, WI 54603 USA; 26 U. S. Department of Agriculture, Southern Research Station, Forest Service, Nacogdoches, TX 75965 USA; 27 Resource Conservation District of the Santa Monica Mountains, Topanga, CA 90290 USA; 28 National Park Service-Santa Monica Mountains Recreation Area, Thousand Oaks, CA 91360 USA; 29 Natural Sciences Division, Seaver College, Pepperdine University, Malibu, CA 90263 USA; 30 U.S. Geological Survey, Southwest Biological Science Center, Tucson, AZ 85719 USA

## Abstract

Changing climate will impact species’ ranges only when environmental variability directly impacts the demography of local populations. However, measurement of demographic responses to climate change has largely been limited to single species and locations. Here we show that amphibian communities are responsive to climatic variability, using >500,000 time-series observations for 81 species across 86 North American study areas. The effect of climate on local colonization and persistence probabilities varies among eco-regions and depends on local climate, species life-histories, and taxonomic classification. We found that local species richness is most sensitive to changes in water availability during breeding and changes in winter conditions. Based on the relationships we measure, recent changes in climate cannot explain why local species richness of North American amphibians has rapidly declined. However, changing climate does explain why some populations are declining faster than others. Our results provide important insights into how amphibians respond to climate and a general framework for measuring climate impacts on species richness.

## Abstract (web summary)

Amphibians have seen large population declines, but the key drivers are hard to establish. Here, Miller et al. investigate trends of occupancy for 81 species of amphibians across North America and find greater sensitivity to water availability during breeding and winter conditions than mean climate.

## Introduction

Global climate change is contributing to significant shifts in the worldwide distribution of species and the structure of local communities^1,2^. Understanding and mitigating the impacts of climate change on biodiversity requires measuring and predicting change across broad geographic, climatic, and phylogenetic scales. As a result, predicting species’ responses to climate changes has become a core objective of conservation biologists^3,4^. Standard approaches to measuring and predicting range shifts largely rely on indirect measures of how changing climate will affect species distributions. Examples include climate envelope approaches that infer future ranges based on current range limits with respect to environmental variables^5^ and biophysical modeling that infers range change based on physiological limitations^6^. In each case, the underlying processes and rate of range shift are inferred rather than measured directly. Range shifts occur because changing climate affects the demographic parameters that control local population sizes (i.e., births, deaths, immigration, and emigration). This calls for directly measuring the population-level processes that underlie population and range-wide responses to climate change. Doing so requires relating variability in climatic conditions directly to measures, such as population growth rate, demographic parameters, or local persistence and colonization probabilities^2,7–11^.

Data limitations have been a hindrance to developing comprehensive demographic research programs to understand climate change responses. An ideal demographic approach might be one that examines individual responses across the complete life-cycle, including age-specific survival and fecundities, as well as movement probabilities. In practice, replicating this effort across multiple populations is costly. As a result, most efforts focus on measuring demographic responses in one or a few populations for one or a few species^12^.

Patch occupancy models offer an alternative and much less data-intensive approach that can capture the key demographic processes that determine changes in distribution^9,13–18^. Dynamic patch occupancy models examine the patterns of occurrence for a species (i.e., whether or not the species is present at a given location and time) and the underlying processes that cause range shifts^19^. Local patterns of colonization and persistence are directly dependent on demographic processes and thus reflect the underlying demographic mechanisms that lead to range shifts^9,19,20^. Examples of the use of patch occupancy models to examine population and community dynamics include models for meta-populations^21,22^ and other single-species models, as well as community models that capture interactions^17,23^ and community responses to change^24–26^. Given the greater ease of collecting data needed to fit these models, they are a pragmatic option for examining demographic responses to climate.

The beginning of the Anthropocene, and its associated changes in land-use and climate, has had profound impacts on the world’s biodiversity^27^. Perhaps no class of species is more emblematic of these global changes than amphibians, which have suffered dramatic losses world-wide over the past four decades^28–31^. Declines appear to be assemblage-wide, such that the loss of one species is not compensated by gain in another—meaning that overall ecosystem function served by amphibians may be reduced^32^. The widespread nature of losses has drawn attention to likely drivers of decline that transcend geographic and taxonomic boundaries.

Changing climate has been suggested as one of the potential drivers of global amphibian declines, either directly (via a direct response to changing abiotic conditions) or indirectly by modifying species interactions^33^, causing hydrological shifts in key breeding habitats^34^, shifting phenology^12,35^, and modifying interactions with pathogens^36^. Numerous studies have demonstrated climate links to demography of individual species, e.g.^11,12,37^, highlighting mechanisms by which a shifting climate can impact amphibian populations. The need to assess the role of climate change on amphibian declines underscores the importance of a multi-scale assessment of climate–demography relationships across amphibian communities.

**Fig. 1:**
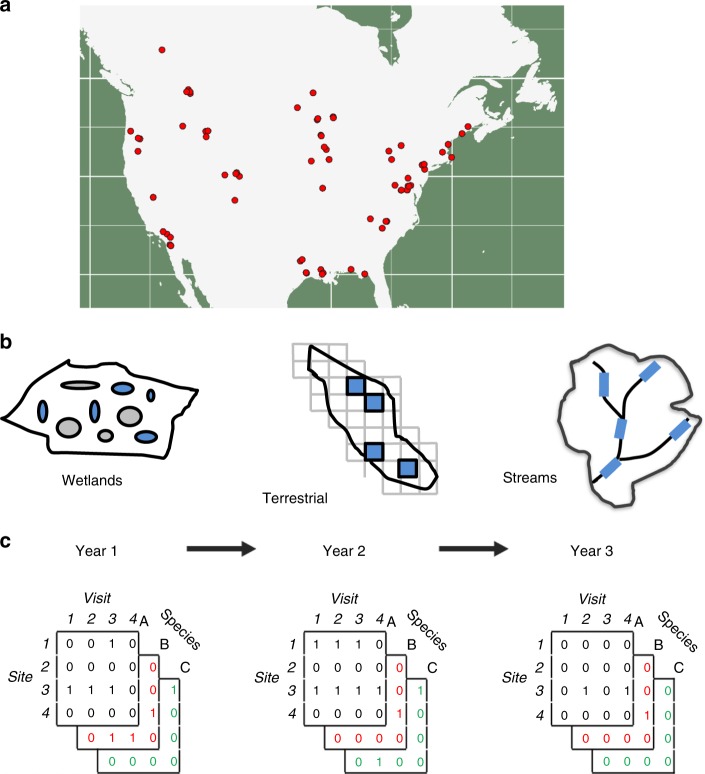
Hierarchical study design. All data used in our study followed a common design. Study areas were located across the United States and Canada (**a**). Individual sites were sampled within each study area, where a site was defined as a waterbody, terrestrial plot, or reach of a stream (**b**). Sites were visited multiple times each year and for multiple years. During each visit one or more species were recorded as detected or not (**c**). Overall we sampled from 86 unique study areas and data included observations for 81 different amphibian species. Maps used in this figure were generated using R package maps

We asked how North American amphibian communities respond to environmental variability using an extensive dataset of long-term amphibian observations, comprised of time-series of detection/non-detection observations of amphibian species. We were able to estimate the direct responses of local populations of amphibians to climatic variability^29,30,38^. We focused on site- specific colonization and persistence probabilities; measures of population response to environmental variability that determine shifts in species distribution^9,13^. First, we show how climatic variability influences occupancy dynamics. We were able to estimate the relationship between annual environmental measures and local colonization and persistence probabilities (Fig. 1). We then used these results to determine how sensitive amphibian populations are to changes in climate^39–41^. We show that sensitivity of species richness to changes in climate for local amphibian communities varies across geographic, climatic, and phylogenetic space. Using this information, we asked if changing climate explains recent North American amphibian declines. The expected rate of change in species richness depends on both how sensitive species are to changes in climate and how much climate has shifted in recent decades. Based on annual records of climatic variables over a 30-year period from 1982 to 2012, we determined whether changes in climate were expected to have caused changes in local species richness.

We demonstrate that year-to-year variation in environmental conditions has strong influences on local colonization and persistence of amphibian populations. The strength of these relationships differs among regions, current climatic conditions, species life history, and taxonomy. Using these estimates we show how sensitivity to changing climate varies across geographic, climatic, and phylogenetic space. Based on the relationships we measure, we find that climate change cannot explain why overall rapid declines are occurring in local species richness. Instead our results suggest that recent climate change both accelerates declines in some regions and buffers losses in other regions.

## Results

### Data collection

**Fig. 2:**
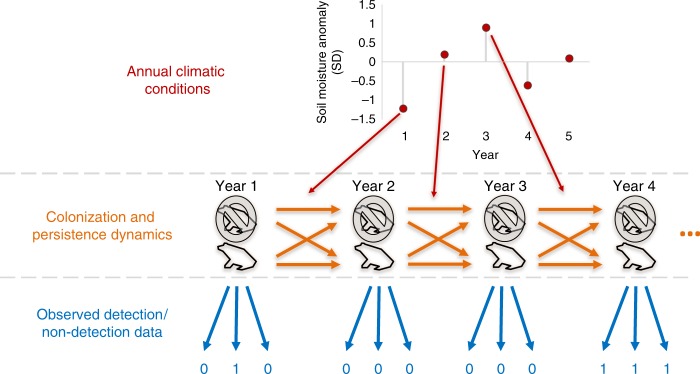
Linking climate variability to local population dynamics. We linked annual variability in climate measures (e.g., winter severity, snowfall, water availability, soil moisture, and extreme summer temperatures) to occurrence dynamics while accounting for observational uncertainty (i.e., the probability of not detecting a species that was actually present during a visit). Our underlying model estimates the relationship between occurrence dynamics (whether a site is occupied in a year conditional on being occupied in the previous year— shown in orange) as a function of annual climate covariates (red arrows). We account for imperfect detection by relating the true occurrence state of sites to actual observed detections and non-detections (zeros and ones—shown in blue) as a function of study area and species-specific detection probabilities. Based on the estimated relationship between annual environmental conditions and transition parameters (i.e., colonization and persistence probabilities), we were able to calculate how sensitive populations are to changes in mean climate

We analyzed time-series data for 81 species of North American amphibians (Supplementary Data 1–3), drawing from 505,387 observations of detection/non-detection data collected at 5370 sites in 86 study areas. All data were collected using a common framework that allowed us to account for study-specific observation error and effort (i.e., rate of false negatives) when estimating changes in local occurrence probabilities (Fig. 1). Our goals were to (1) quantify the relationship between climate and local population dynamics and factors that determine these relationships, and (2) assess whether the overall declines and variation in the rate of decline in North American amphibians could be explained by recent changes in climate. Recognizing the limitation of static approaches to inferring climate associations, we used an approach to quantify the climatic processes directly affecting the population level processes that determine species’ ranges shifts (i.e., local colonization and persistence)^2,7–9,42^. We focused on how colonization and persistence of local populations were related to annual variation in five climate variables thought to affect key components of amphibian life-cycles: winter severity, snowfall, breeding water availability, summer soil moisture, and maximum temperature (Fig. 2). In so doing, we directly measured how climate drivers are affecting the processes that determine range shifts, avoiding equilibrium assumptions inherit to static modeling approaches^9^.

### How does climatic variability affect occupancy dynamics

**Fig. 3:**
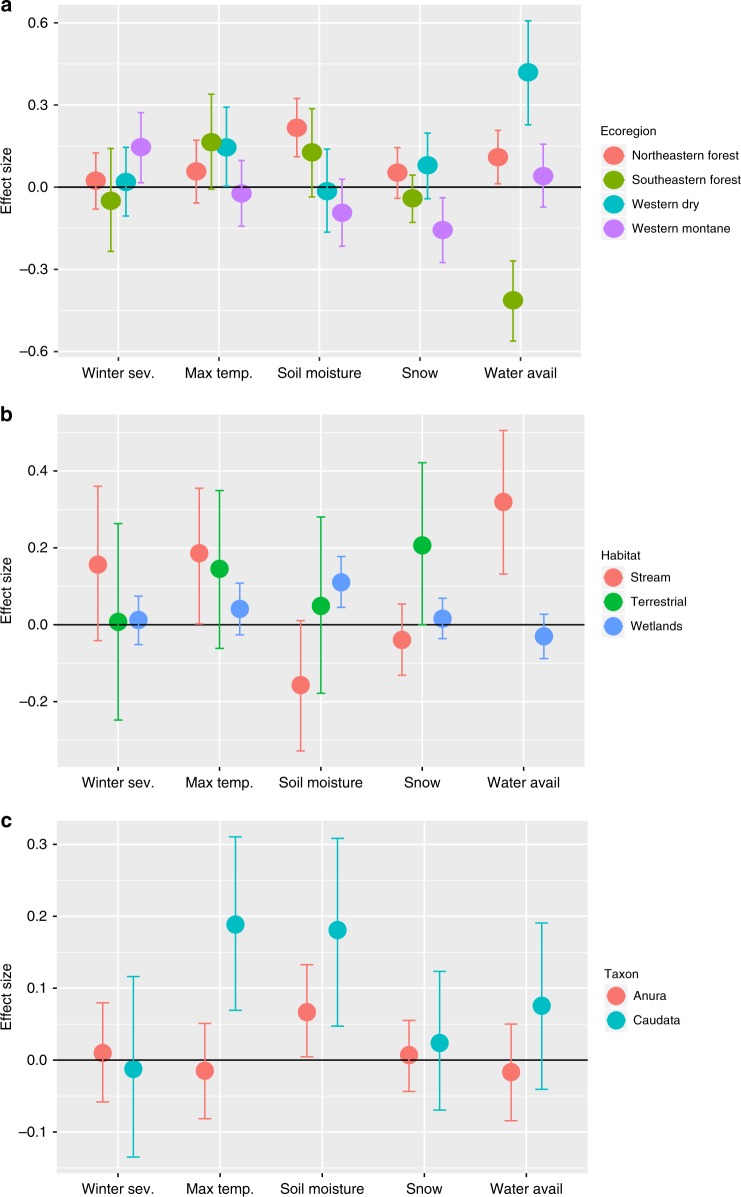
Ecoregion, site, and taxonomic differences in the relationships of climate variability to colonization and persistence probability. We estimated the relationship between annual conditions for five climatic drivers and species turnover and how it varies among ecoregions (**a**), site type (**b**), and taxonomic grouping (**c**). Effect size is on a logistic scale and represents the effect of a 1-SD change in the climate covariate on colonization and persistence. Error bars are the 95% credible interval for the estimate. Positive values indicate a location is more likely to be occupied in years after an increase in the climate covariate (higher colonization and persistence probability), whereas negative values are associated with declines in years with higher values. Plotted effect sizes for categorical variables are the group mean effects. For example, greater winter severity, as measured by higher values of the air-freezing index, was associated with increased colonization and persistence for species in the Western Montane region. Estimates and SE for effect sizes can be found in Table 1

**Fig. 4:**
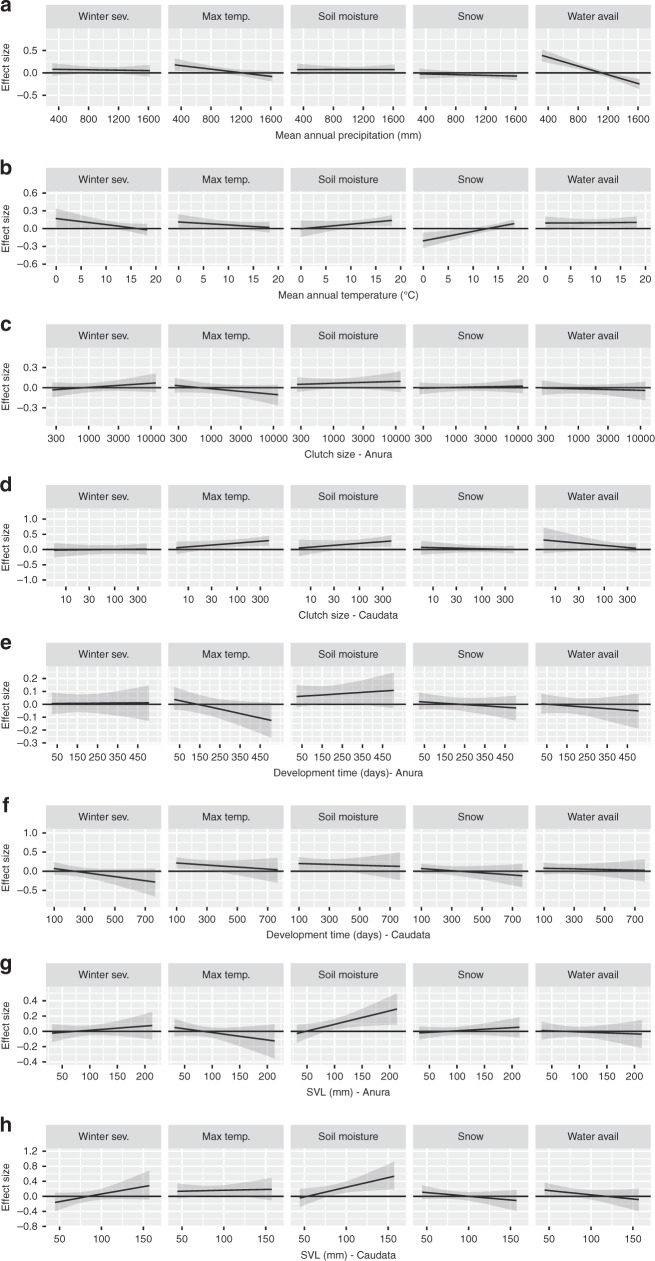
Climatic and life history differences in the relationships of climate variability to colonization and persistence probability. We estimated the relationship between annual conditions for five climatic drivers and species turnover and how it varies with respect to current climate (**a**, **b**) and life history traits (**c**–**h**). Effect size is on a logistic scale and represents the effect of a 1-SD change in the climate covariate on colonization and persistence. Error bars are the 95% credible interval for the estimate. Positive values indicate a location is more likely to be occupied in years after an increase in the climate covariate (higher colonization and persistence probability), whereas negative values are associated with declines in years with higher values. Plotted relationships for continuous variables show how the effect size of a given climate variable on colonization and persistence changes as the covariate changes. For example, greater water availability during breeding had a positive association with occupancy in the following year in sites with low mean annual precipitation and a negative effect in sites with high mean annual precipitation. Estimates and SE for effect sizes can be found in Table 1

We first determined how annual colonization and persistence probabilities were related to annual anomalies for each of our five climate variables (Table 1, Figs. 3 and 4, Supplementary Figs. 1 and 2). Key drivers of local occurrence dynamics differed among the four ecoregions we examined (i.e., northeastern forest, southeastern forest, western dry, and western montane). Average colonization and persistence was highest in years following summers with high soil moisture for amphibians in northeastern forest communities. A similar positive effect occurred following years with higher water availability during breeding in western dry and northeastern forest communities, while greater water had a negative effect on colonization and persistence in southeastern forest communities. Our two winter measures, winter severity and snowfall, had their greatest effect on western montane amphibians. Increased water availability had the greatest positive effect on colonization and persistence for locations with lower mean annual precipitation, while increasing snowfall had the most negative effects in colder locales. Within salamanders (Caudata) there was a general positive effect of summer temperatures and soil moisture on colonization and persistence probabilities. In general, life-history variation was not a strong predictor of how climatic variation affected occurrence dynamics. The exception was that soil moisture had a greater positive effect on colonization and persistence in species with larger body sizes, both in Anura and Caudata.

**Table 1:** Estimated Relationships between annual conditions and species turnover

Factor	Level	Climate variables				
		Winter severity	Maximum temperature	Soil moisture	Snow water equivalent	Water availability
Eco-region	W. dry	0.019 (0.065)	0.145 (0.074)	−0.015 (0.076)	0.080 (0.061)	**0.419 (0.096)**
	W. montane	**0.146 (0.065)**	−0.023 (0.061)	−0.093 (0.062)	**−0.156 (0.060)**	0.041 (0.058)
	NE forest	0.024 (0.052)	0.057 (0.059)	**0.217 (0.054)**	0.052 (0.047)	**0.110 (0.050)**
	SE forest	−0.050 (0.095)	0.164 (0.088)	0.127 (0.083)	−0.41 (0.044)	**−0.413 (0.074)**
Habitat	Wetland	0.012 (0.033)	0.041 (0.034)	**0.110 (0.034)**	0.016 (0.027)	−0.030 (0.029)
	Stream	0.156 (0.103)	**0.186 (0.090)**	−0.157 (0.087)	−0.039 (0.047)	**0.319 (0.095)**
	Terrestrial	0.008 (0.129)	0.145 (0.104)	0.049 (0.115)	**0.206 (0.107)**	<><>
Climate	Mean precip.	0.007 (0.039)	−0.064 (0.035)	−0.024 (0.034)	−0.013 (0.028)	**−0.173 (0.036)**
	Mean temp.	−0.048 (0.043)	−0.026 (0.037)	0.054 (0.035)	**0.097 (0.031)**	−0.011 (0.033)
Taxa	Anura	0.017 (0.034)	−0.022 (0.035)	0.049 (0.034)	0.009 (0.027)	−0.025 (0.032)
	Caudata	−0.003 (0.065)	**0.178 (0.060)**	**0.175 (0.067)**	0.019 (0.052)	0.078 (0.059)
Caudata—life history	Clutch size	0.025 (0.68)	0.107 (0.065)	0.096 (0.074)	−0.019 (0.065)	−0.138 (0.104)
	Dev. time	−0.089 (0.070)	−0.056 (0.056)	−0.012 (0.059)	−0.074 (0.056)	−0.029 (0.054)
	Size (SVL)	0.158 (0.115)	0.000 (0.086)	0.178 (0.103)	−0.096 (0.085)	−0.080 (0.091)
Anura—life history	Clutch size	0.060 (0.054)	−0.067 (0.057)	0.021 (0.058)	0.012 (0.040)	−0.024 (0.058)
	Dev. time	0.025 (0.036)	−0.067 (0.037)	0.006 (0.035)	−0.017 (0.025)	−0.015 (0.035)
	Size (SVL)	0.037 (0.032)	−0.040 (0.038)	**0.067 (0.036)**	0.006 (0.024)	−0.012 (0.035)

We estimated the relationship between annual conditions for five climatic drivers and species turnover, and how it varies among regions and with respect to long-term climate normals, habitat, taxonomic groupings, and species traits. Effect size is on a logistic scale and represents the effect of a 1 SD change in the climate covariate on colonization and persistence. Precision of estimates is given by the standard deviation of the posterior and is shown in parentheses. Positive values are associated with higher colonization and persistence values after years where the climate variable was above the long-term mean. For example, we found a positive effect of more severe winters on colonization and persistence in western montane sites. Thus, we would expect local species richness to decline in response to warming winters due to decreased occupancy. Relationships are also plotted in Fig. 3. *Note*: Effect sizes that did not include 0 in the 95% credible interval (CI) are denoted in bold.

### How sensitive are amphibians to changes in climate

**Fig. 5:**
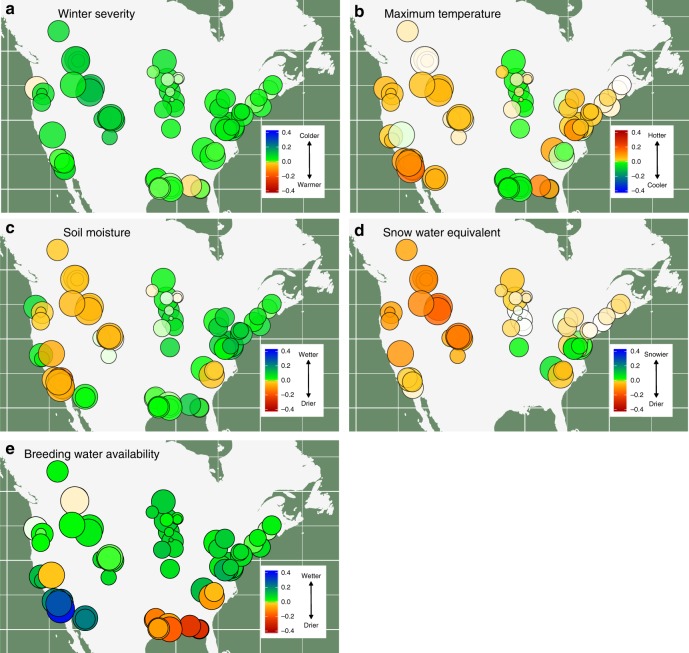
Sensitivity to climate change across geographic space. We estimated how sensitive species richness was to changes in mean climatic conditions based on the estimated relationship between annual climate measures and colonization and persistence probabilities. Sensitivity is the expected rate of change in local species richness of individual sampling locations with respect to changes in the climatic variables. For example, a value of −0.1 would indicate a one standard deviation increase in the climate variable would lead to a 10% loss in species richness. All climate variables are standardized based on observed variability over a 30-year period in the variable, allowing for direct comparison among climate variables. Positive values occur when we estimated a positive change in species richness (i.e., mean species occurrence probabilities for a location) as values of the climate variable increased (i.e., more severe [i.e., cold] winters (**a**), warmer extreme summer temperatures (**b**), greater snowfall (**c**), greater soil moisture (**d**), and more water during and immediately prior to breeding (**e**)). Negative values represent a predicted decline in local species richness with a shift to higher values of the climate variable. Cool colors (greens and blues) denote that the species richness responds positively to either cooler or wetter conditions, depending on the climate variable. Warm colors (yellow, orange, red) denote that species richness responds positively to warmer or drier conditions for the climate variable. Circle sizes are proportional to the sample size for the study area (number of unique species, site, and year combinations). Missing values occur for snow water equivalent and winter severity for warmer areas where snowfall and days with mean temperature less than 0 were minimal. Maps used in this figure were generated using R package maps

**Fig. 6:**
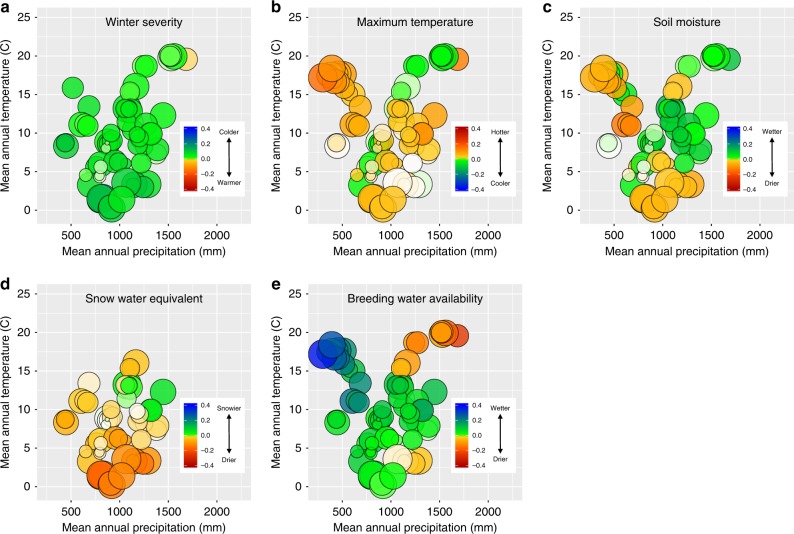
Sensitivity to climate change across climatic space. We estimated how sensitive species richness was to changes in mean climatic conditions based on the estimated relationship between annual climate measures and colonization and persistence probabilities. Positive values occur when we estimated a positive change in species richness (i.e., mean species occurrence probabilities for a location) as values of the climate variable increased (i.e., more severe [i.e., cold] winters (**a**), warmer extreme summer temperatures (**b**), greater snowfall (**c**), greater soil moisture (**d**), and more water during and immediately prior to breeding (**e**)). Negative values represent a predicted decline in local species richness with a shift to higher values of the climate variable. Cool colors (greens and blues) denote that the species richness responds positively to either cooler or wetter conditions, depending on the climate variable. Warm colors (yellow, orange, red) denote that species richness responds positively to warmer or drier conditions for the climate variable. Circle sizes are proportional to the sample size for the study area (number of unique species, site, and year combinations). Missing values occur for snow water equivalent and winter severity for warmer areas where snowfall and days with mean temperature less than 0 were minimal. We did not measure the effect of breeding water availability for species only measured in terrestrial plots, where surveys were not timed with peak breeding activity

**Fig. 7:**
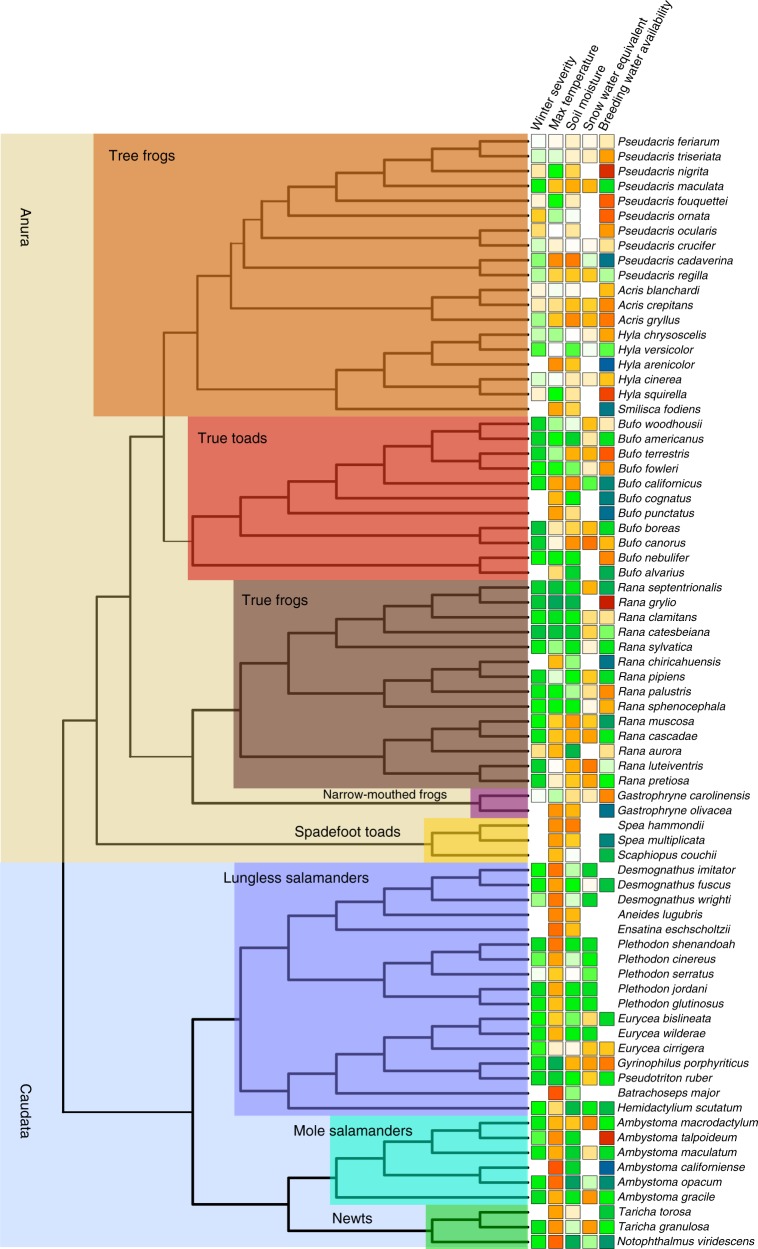
Sensitivity to climate change across taxonomic space. We estimated how sensitive species richness was to changes in mean climatic conditions based on the estimated relationship between annual climate measures and colonization and persistence probabilities. Positive values occur when we estimated a positive change in species richness (i.e., mean species occurrence probabilities for a location) as values of the climate variable increased. Cool colors (greens and blues) denote that the species richness responds positively to either cooler or wetter conditions, depending on the climate variable. Warm colors (yellow, orange, red) denote that species richness responds positively to warmer or drier conditions for the climate variable. Missing values occur for snow water equivalent and winter severity for warmer areas where snowfall and days with mean temperature less than 0 were minimal. We did not measure the effect of breeding water availability for species only measured in terrestrial plots, where surveys were not timed with peak breeding activity

Using a global model that combined predictors from the previous analysis, we estimated how sensitive local species occurrence and species richness were to changes in each of our climate variables (Figs. 5–7). Sensitivity is the expected rate of change in equilibrium occurrence probabilities (i.e., the proportion of sites occupied by a species) as annual values for each of our climate variables increase. The measure integrates the effects of climate on colonization and persistence, as measured in the last section, along with the effect of changes in colonization and persistence on expected number of locations where a species is expected to occur. A positive sensitivity means that individual species occurrence or, when averaged across species in a local community, overall species richness will increase with a positive directional shift in the associated climate measure.

Examining patterns of sensitivity suggests there are strong geographic and climatic patterns in how sensitivity to climate change is structured (Figs. 5 and 6). Colder climates and western regions, especially montane sites, were most sensitive to changes in winter conditions. Both warmer winters and greater snowfall had negative impacts on local species richness. In contrast to a negative effect of winter warming, warmer summer temperatures had a positive effect on local richness in almost all locations except in the mid-continent. Not surprisingly, amphibians also exhibited generally positive responses to increases in both breeding water availability and soil moisture across most locations. However, the magnitude of the sensitivity to changes in each was strongly dependent on local climate. For both breeding water availability and soil moisture, the strongest positive effects occurred in sites with highest mean annual temperatures. The one region where breeding water availability had negative effects on richness was the southeast, where recent extreme rainfall events may be having a negative effect on breeding populations^33^.

Our results suggest genera may respond differently to changing climate. Warming winters were again expected to have generally negative effects across species. However, the negative effects of winter warming were much more muted or switched to being positive for the tree frogs (Fig. 7). Warmer summers on the other hand were expected to benefit all salamander species except for *Gyrinophilus porphyriticus* and *Pseudotriton ruber*, both of which are large-bodied stream dwelling salamanders, and generally restricted to cold, headwater streams which may be more sensitive to warming than other habitats. The strongest positive responses to warmer summers were observed for mole salamanders and newts. For other covariates that relate to water availability, effects did not show a consistent phylogenetic pattern and instead were more strongly related to spatial variation. This suggests that local factors, such as topography, hydrology, and geomorphology may be the primary predictor of how changes in precipitation and moisture affect amphibian communities.

### Does changing climate explain amphibian declines

How climate change affects the distribution and local richness of species will depend on how sensitive species are to climate change, as well as how quickly climate is changing. We only expect to see changes in species richness when the two align. Using historical climate records, we estimated the expected rate of change in occupancy that could be attributed to climate alone as predicted by our dynamic species occurrence model for the 30-year period from 1983 to 2013. We estimated that the average rate of annual decline across all populations that could be attributed to changes in climate would be −0.14% (95% credible interval: −0.19% to −0.09%), with 37% of populations expected to increase. The results were similar using an alternative method, where expected decline was directly modeled using the estimated annual occurrence dynamic parameters (−0.26% mean annual decline expected; 41% of populations expected to increase). This stands in contrast to an observed −3.4% mean annual rate of decline across all the species and locations in our data set (−4.6% to −2.2%). While by no means conclusive, our model and associated climatic variables do not explain why, on average, amphibians are rapidly declining in North America and suggests that other causes are to blame. In addition to looking at overall expected rate of decline over the 30-year period, we also examined whether the expected trend was correlated with conservation status. We found no evidence for differences between IUCN Red List species vs. species of least concern (*p* = 0.20, *R*^2^ = 0.004, Supplementary Data 3).

**Fig. 8:**
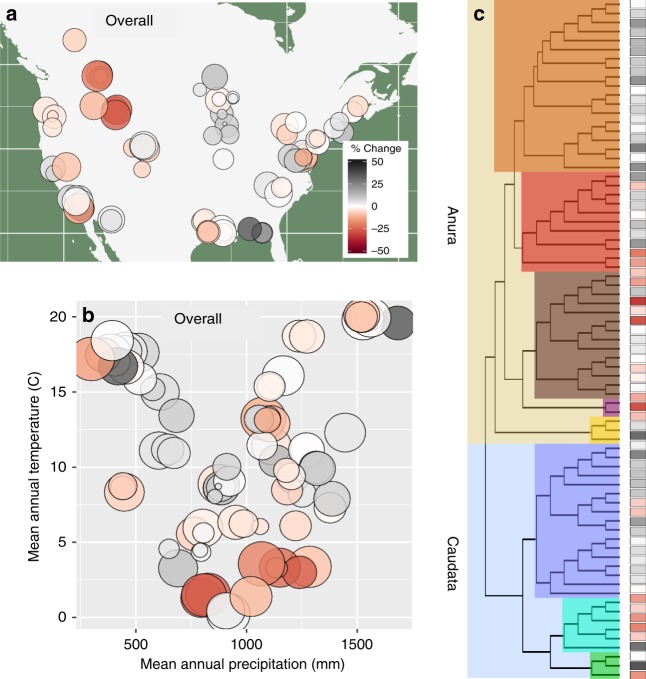
Estimated effects of recent climate change on local species richness. We estimated the change in local species richness based on recent changes in mean climate from 1982 to 2012. Locations where our analysis indicates that declines were expected to have occurred are denoted using a red color scale, while areas where increases are expected to have occurred are denoted using a gray color scale. Circle sizes are proportional to the sample size for the study area (number of unique species, site, and year combinations). Values are plotted in geographic (**a**), climatic (**b**), and phylogenetic space (**c**). Expected change with respect to individual changes in each of the five climate drivers is shown in Supplementary Fig. 3. Maps used in this figure were generated using R package maps

While climate does not explain why average declines are so steep, we found that the expected rate of climate-driven change in occurrence from our model was correlated with observed trends (*β* = 6.6, 1.4–11.7; *r* = 0.44). Thus, we found a match between where our model predicts climate change should be leading to local losses of species due to recent climate change, and the locations and species that are currently exhibiting the fastest rate of decline. This suggests that climate change is exacerbating declines in some areas and buffering declines in others. We estimated that climate change is having the greatest negative effect in regions including the Northern Rocky Mountains, parts of southern California and Arizona, and in the southern Mississippi Delta region (Fig. 8). This geographic pattern matches a previous analysis that shows the strongest declines are occurring in the Rocky Mountain and Western regions^30^. Local species richness would be predicted to increase in the Midwest and Northeast if climate alone were acting on range dynamics and may explain why overall amphibian decline is slower in these regions. Our analysis suggests that though it may be an important factor in some populations, climate is not the primary factor driving continental-scale population declines^30^. Decomposing the expected trend to examine the predicted effect based on each climate variable treated individually suggests that changes in summer temperature and winter severity are primary drivers of contemporary change (Supplementary Fig. 3).

## Discussion

Our study offers one of the most comprehensive analyses, in terms of geographic-extent, climatic-extent, and phylogenetic-extent, of the direct effects of current environmental variability on local population dynamics (i.e., annual probabilities of local persistence and colonization). Using a community-modeling approach, we were able to quantify how species richness of North American amphibians has responded to recent climate change, and how sensitive communities are to future changes in climate. As hypothesized, sensitivity to climate varied based on region, local climate normals, habitat type, and, to a lesser extent, species life history traits. For some variables, such as warmer winter temperatures and decreased water availability during the breeding season, we measured a near universal negative effect on local species richness. For other climatic variables, the observed response differed widely across geographic, climatic, and phylogenetic space, as has been found in other taxonomic groups^11,43,44^.

Our results identify populations that will be most sensitive to specific types of climate change, and provide predictions regarding the mechanisms underlying among-population differences in responses to changing climate. For example, we found that summer drought has the strongest impact in warm and dry regions, decreased snowfall has a smaller effect in eastern compared to western North America, and winter warming is predicted to decrease species richness for all species groups except tree frogs.

While we demonstrate that amphibian communities are sensitive to changes in climate, our results suggest that changing climate is not the proximate cause for ongoing assemblage-wide declines that have been observed in North American amphibians. Instead, we find that recent change in climate is a strong predictor of why local species richness is declining more quickly in some regions and that positive effects of climate may be buffering declines in other regions. For 37% of studied locations, we predicted that local species richness would have increased if climate was the primary factor determining changes in amphibian communities. We cannot eliminate factors we have not tested here. However, it would be surprising to find so many climate “winners” in our analysis if climate was a primary driver of the severe declines being observed in North American amphibian populations.

While our conclusions apply to contemporary declines (i.e., changes in the previous 30 years), this does not preclude that continental rather than just regional impacts on species richness will occur in the future as species are exposed to conditions outside of the range of variation measured in our study^45^. However, current rates of change act more strongly on the composition of species richness and on regional species pools rather than affecting universal changes^46^. At the same time, high rates of turnover may be especially detrimental to species that are rare or have restricted ranges^47^, although we find no correlation in contemporary effects and IUCN status.

Expected responses to changing climate were context specific, with a range of observed sensitivities to each of our climatic variables. For example, water availability during breeding predictably had the greatest benefit in areas where the mean climate was hot and dry. Alternatively, the effect of winter warming appears to have very different effects, depending on the taxonomic groups in which responses are measured.

Our results highlight the importance of winter conditions in determining population and community responses, especially in colder climates. This result is consistent with other recent studies^10–12,44^ that suggest winter and below-ground ecology is important in determining how species will respond to climate change. In general, we saw greater negative effects of winter warming, while warming during the summer tended to have positive impacts on species richness. The positive effect of warmer summers suggests an interesting hypothesis related to immune function. Amphibians and other ectotherms are limited in their ability to maintain the higher body temperatures needed to mount an immune response to pathogens^48^. Higher extreme temperatures could indirectly benefit populations by increasing the ability to fight infections. This may be especially important as novel pathogens, such as chytrid fungus (*Batrachochytrium dendrobatidis*) and *Ranavirus* are having negative effects on many North American amphibian populations^49–52^.

The dynamic species occurrence model we employed is a promising approach for studying other communities where multi-year data sets are available. The analysis relies on a simple data structure for sampling (how many times a species was detected over multiple sampling visits) and readily accounts for study-specific species detection differences when capturing the basic population level processes determining range shifts. As a result, we were able to directly measure how the dynamics of local amphibian populations were related to variation in climatic variables. This stands in contrast to standard space-for-time assumptions that are the back-bone of most large-scale analyses. Our results provide new, foundational information from which to build specific hypotheses about mechanisms behind differing species’ responses to changes in climate.

At the same time, the degree of complexity that we were able to measure in the hypothesized relationships between local dynamics and climate had limits. Even with a large data set, we were unable to consider non-linear, threshold, or extreme value effects, or how factors such as range position or degree of isolation affect responses. Future work should delve further into the complexities of these observed responses. Achieving this will require even greater spatial coverage and longer-time series and underscores the value of long-term data, collected using systematic and repeatable methods, and the importance of cooperation and data-sharing among programs and individuals.

## Methods

### Data

We aggregated data from long-term studies of amphibian communities conducted throughout the United States and Canada. Our overall data set followed a nested hierarchical design, including data across a series of distinct study areas within each of which we collected observations for multiple species, at multiple sites, and across multiple years at repeated intervals within years (Fig. 1). We limited data to studies that lasted a minimum of 3 years and where sites were visited multiple times within a year to collect observations of whether each species was detected or not. We included data through 2013, the latest year all climate data used in the analysis was available. In some cases, study areas spanned a large geographic area. If a study area included sites that occurred within multiple HUC-4 designation watersheds, we treated sites in each watershed as a separate study area for the purposes of analyses. Designating study areas in this way allowed us to control for the hierarchical nature of data collection (i.e., study areas designate multiple non-independent sites occurring within a local region)^38^.

All studies included in this analysis used a shared data collection framework that required making repeat visits within a defined period in each year, where the target species were recorded as detected or not detected. This sampling design allowed us to estimate a common metric across every study, the probability a site was occupied in a given year, while using repeated visits to estimate and control for uncertainty that occurs when species that may be present go undetected. This was accomplished by using statistical models that accounted for incomplete detection when generating estimates of local occurrence and turnover^13,53^. The ability to control for differing detection probabilities was crucial in allowing us to include data collected using multiple observation methods, and which varied in sampling effort and ability to detect species given the environment and sampling method. Field methods used for observing species were chosen to be appropriate for local conditions, and included visual encounter surveys, minnow traps, dip-nets, cover-object surveys, pitfall traps, drift fences, human auditory call surveys, and automated auditory recording devices.

For lentic surveys we defined a site as an individual water-body, for lotic surveys we divided streams into segments (range from 30 to 200 m in length), while we defined sites for non-aquatic terrestrial surveys as a block of area for which a visual encounter or cover object survey occurred (10 by 10 m in most cases) or the effective trapping area of a pitfall array. Lentic and lotic surveys occurred during the annual breeding period of each species, while terrestrial surveys occurred when animals were most likely to be active on the soil surface and thus available for detection. Again, by relying on repeated visits to account for different detection probabilities among species, study areas, and survey methods we were able to estimate occupancy states for all locations in the database.

### Statistical model

We estimated parameters using a hierarchical dynamic site occupancy model, which accounted for observational uncertainty (i.e., false absences) to estimate annual occurrence probability as a function of whether a site was occupied in the previous year and the effect of annual variation in climatic variables (Fig. 2)^13,54^. We accounted for variation in parameters among sites and species using a hierarchical design, employing hyper-parameters (i.e., random-effects) to account for variation among sites and dependence of sites within study areas and within species^24,38,55^.

Our occupancy model used the auto-logistic formulation^15^. We estimated *ψ*_*ijk*,*t+1*_ the probability that the *k*th species occurred in the *i*th site in the *j*th study area at time *t* + 1 as a function of: (1) a study area and species-specific random-intercept, *α*_*jk*_; (2) a species and study area-specific random-effect for whether the site was occupied in the previous time step *δ*_*jk*_; and (3) a response vector, **β**_*x*,_ and covariate vector, **X**_*ijkt*_ related to annual weather values and factors thought to interact with climate to affect turn-over. The hierarchical model took the form of:

1 $${\mathrm {logit}}\left( {\psi _{ijk,t + 1}} \right)\sim \alpha _{jk} + \delta _{jk} \times z_{ijkt} + {\mathbf{\beta }}_x \times {\mathbf{X}}_{ijkt},$$

2 $$\alpha _{jk}\sim {\mathrm{Norm}}\left( {\mu _\alpha ,\sigma _\alpha } \right),$$

3 $$\delta _{jk}\sim {\mathrm{Norm}}\left( {\mu _\delta ,\sigma _\delta } \right).$$

If the site was occupied in the previous year, *z* = 1, and if the sites was unoccupied, *z* = 0. This approach allowed us to simultaneously estimate the effects of annual climate on site colonization, *γ* (i.e., the probability a site not occupied in year *t* − 1 is occupied in year *t*), where

4 $$\gamma _{ijkt} = \alpha _{jk} + {\mathbf{\beta }}_x \times {\mathbf{X}}_{ijkt},$$

and persistence, *φ* (i.e., the probability a site occupied in year *t* − 1 remains occupied in year *t,* this is also equivalent to 1—probability of local extinction), where

5 $$\varphi _{ijkt} = \alpha _{jk} + \delta _{jk} + {\mathbf{\beta }}_x \times {\mathbf{X}}_{ijkt}.$$

This allowed our model to capture the local population dynamics that lead to change in species distribution and local richness. Initial occupancy was treated as a random-effect that varied among different combinations of species and study area.

We accounted for detection by incorporating a basic observation component to the model where detection probability (*p*_*j*_, the probability of detection conditional on the species being present) varied among combinations of study area, species, and time, so that:

6 $${\mathrm{logit}}\left( {p_{jkt}} \right)\sim {\mathrm{Norm}}\left( {\mu _p,\sigma _p} \right).$$

We used vague priors for all parameters. All models were fit using JAGS^56^ and called using the R2jags^57^ package in R^58^. After 5000 iterations of burn-in, we ran 20,000 iterations, saving every 10th value to estimate the posterior. Three chains were run for each model and we checked for convergence based on the Gelman–Rubin statistic (*R* < 1.05)^59^.

### Climate variables

We focused on a set of climate/weather variables that we chose to represent potential abiotic stressors that occur during specific periods of the amphibian life-cycle (breeding, non-breeding, and over-wintering). Focusing on this specific set of parameters reduces the chances of Type I errors, while allowing us to focus on the species, site, and regional factors that lead to variation in climate sensitivity for a core set of climatic drivers. Limiting our analysis to these five variables does not preclude that other measures of climate are also important in determining occurrence dynamics of amphibians. However, we believe these variables capture a major set of environmental drivers during key life history stages. Each of the variables we assessed was standardized to have a mean of 0 and standard deviation of 1 for each site based on values from the previous 30 years. Standardizing variables in this way ensured that estimated effect size was relative to the actual amount of variability in that climate variable at that site. Estimated slopes from the models therefore measure the response of colonization and persistence to a 1 SD change in each of the climatic drivers. By standardizing based on local variability, we ensure that measures of response are relative to local conditions. In addition, some of our climate measures, such as for winter severity, differ greatly in magnitude while others already come from data sources that standardize based on local conditions. Making sure all are standardized ensures that differences in sensitivity are not due to differences in local magnitude of the measurement.

To measure winter severity, we used the air-freezing index (AFI) as a single index of overall winter severity. The AFI captures the overall length and magnitude of cooling during winter months. AFI is calculated by taking the cumulative sum of temperatures from August 1 to July 31 for a year and calculating the differences between the fall peak and spring minimum for the cumulative values as a measure of overall temperature deficit^60^. AFI is a measure of overall potential freezing capacity due to atmospheric temperatures. Because most species spend the winter in the sub-surface below the frost-line or in water that does not freeze completely, the AFI should be a good proxy of relative freezing depth and the length of time over which surface soils will be frozen. The actual freeze depth will depend on local soil characteristics as well as snow depth, which acts to insulate soil from colder air temperatures. Calculations were made using the daily average temperature from the PRISM climate data set (PRISM Climate Group, Oregon State University, http://prism.oregonstate.edu, accessed 19 August 2014). This variable was only included for sites where freezing regularly occurred (mean annual AFI > 20).

As a proxy for potential heat stress we calculated the highest 10-day moving average for mean temperature for each site. We used this averaged metric rather than a single maximum temperature value because most amphibians spend the majority of time either in water or under the soil surface during the hottest periods of the year, creating a moderating effect, where realized temperatures are the product of averages over an extended period of time^61^. Calculations were made using daily average temperature from the PRISM climate data set (PRISM Climate Group, Oregon State University, http://prism.oregonstate.edu, accessed 19 August 2014).

We used average relative snow water equivalent (SWE) during winter months as an index of snow conditions. The index was derived from a multi-model ensemble prediction of relative SWE from eight soil-moisture models^62^. The model predicts the relative snowfall on a monthly basis, measured in quantiles of the long-term distribution. We averaged values for the winter months (December–April) to derive an overall snowfall index for a given winter. This variable was only included for sites where winter temperatures were sufficiently cold to allow for extended snow accumulation (mean annual AFI > 200).

We hypothesized that availability of water would be influential for breeding success of most pond and stream breeding amphibians. To capture this, we used a short-term measure of water-balance, the standardized precipitation and evapotranspiration index (SPEI) calculated over a 3-month period ending in the month for which peak reproductive activity occurred for each of the species in each of the study areas. Previous work suggests short-term measures of water-balance predict annual differences in wetland inundation for many of the study areas^10,33,34,38,42,63^. We did not include this predictor for terrestrial sites because the metric was chosen primarily for its potential to affect dynamics of water-bodies. In addition, timing of peak breeding is poorly characterized for many of our terrestrial amphibian species.

Summer is a period when many species occur in terrestrial environments and have a high potential for stress from desiccation. As a proxy for desiccation potential, we used an average measure of soil moisture over the period of June 15–August 31 in each of the study areas. The measure was derived from an ensemble model that averaged results of eight soil-moisture models and captures drought conditions well^62^.

### Variables affecting climate relationships

We were interested in determining how responses to each of our five climatic drivers varied across species, sites, and regions. We considered the following variables, which we expected would influence how sensitive amphibians were to directional shifts in climate.

We included a number of species-specific factors in models. We examined general taxonomic differences between Anurans (frogs and toads) and Caudates (salamanders and newts), as well as differences related to life history traits within each of the broad taxonomic groups. These traits included average size (mean snout–vent length at maturity), average clutch size (log-transformed), and development time (mean length of tadpole or larval stage). We compiled these species-specific values in a life-history database using existing literature and reference texts for each of the species in the database.

In 10% of cases, life history data were missing for one of the three traits we focused on in our analyses. We imputed values using a phylogenetic factor analysis model where observed traits are assumed to arise as linear combinations of two phylogenetically correlated latent factors which evolve via a simple Brownian trait evolution model^64^. The latent factors are drawn from independent mean zero multivariate normal distributions with covariance matrix ∑, a *K* by *K* covariance matrix with unit variance and non-diagonal elements equal to the product of scaled phylogenetic distance and *λ*, which represents the degree of phylogenetic signal estimated from the data. When *λ* = 1, the correlation structure of the latent factors is consistent with a Brownian motion, and when *λ* = 0, ∑ becomes an identity matrix and there is no phylogenetic autocorrelation in the latent factors. A triangular loading matrix relates the latent factors to the observed traits, which are observed with error, representing correlations among traits as a consequence of shared responses to the latent factors^64^. We sampled from the posterior distribution of model parameters using Hamiltonian Monte Carlo fit using the Stan software package and used the posterior mode of the predicted missing trait values as a point estimate for trait imputation^65^. In this way, imputations incorporate information from two sources: (1) trait data from closely related species and (2) incomplete trait data for the species whose traits are being imputed. To determine phylogenetic distance among neighbors, we constructed a posterior consensus tree in MrBayes^49^ using previously published sequence data^50^ that was subsetted to include just the species in this analysis.

In addition, we included measures related to site characteristics. Sites were classified as wetland (lentic), stream (lotic), or terrestrial (non-aquatic).

We also classified each study area into one of four broad eco-regions. These regions were chosen because they relate to broad-scale biogeographic, climatic, and physiographic differences found in North American amphibian habitats. Our regional classifications combine level-I and II eco-region designations for North America. Study areas were classified as: (1) western montane—higher elevation western sites belonging to the northwestern forested mountains ecoregion designation; (2) western dry—the combination of desert, grassland, and Mediterranean sites belonging to the great plains, North American deserts, Mediterranean California ecoregion designations; (3) northeastern forest—sites in forested areas in the east including the northern forest ecoregion and the mixed wood plains and Ozark, Quachita, Apalachian forests within the eastern temperate forest ecoregion, and (4) southeastern forest—more southern sites in the east, including the southeastern USA plains and Mississippi Alluvial and southeast USA coastal plains within the eastern temperate forest ecoregion. A small number of study areas in the Pacific Northwest did not fit into this classification. There was not enough replication to consider it as its own category so these sites were treated as an ‘Other’ category and not reported in results related to the factors influencing climate sensitivity.

We also were interested in how climate sensitivity depended on long-term climate normals. We considered mean annual temperature and mean annual precipitation for a site. 30-year normals were calculated using PRISM data for mean temperature and total precipitation from 1982 to 2012 (PRISM Climate Group, Oregon State University, http://prism.oregonstate.edu, accessed 19 August 2014).

### Analysis

We determined the relative influence of annual variation in each of the climate variables on local turnover probabilities and how this was related to each of the species, site, and regional factors. Our hierarchical occurrence model (Eq. (1)), included main effects for each of the five climate variables (i.e., site-specific annual anomalies) and the interaction between the climate variables and each of the species, site, and regional factors described in the previous section. The estimated effects (**β**_*x*_) were a measure of the influence of climate on the dynamic parameters (site colonization *γ* and persistence *φ* probabilities).

Using the estimated relationships between annual climate variables and amphibian turnover dynamics, we predicted how occurrence probabilities would change with shifts in mean climate values. We define sensitivity as the expected change in species richness associated with a specified change in mean climate values. This value, when averaged across a set of species, also reflects the expected change in species richness associated with a specified change in mean climate values. We calculated the sensitivity of the expected equilibrium occupancy probabilities (ψ^*^) to changes in the mean value of each of our climate predictors^39^. We measured sensitivity as a rate of change in the relative occupancy with respect to changes in each of the five standardized climate variables, denoted as X^*^. Using the chain-rule we were able to calculate the full sensitivity for a given species and study area as

7 $$\frac{{{\mathrm {d}}\psi }}{{{\mathrm {d}}X^ \ast }} = \frac{1}{{\psi ^ \ast }}\mathop {\sum}\limits_{T{\it{\epsilon }}{\mathrm{\gamma }},\phi } {\frac{{{\mathrm {d}}\psi ^ \ast }}{{{\mathrm {d}}T}}} \frac{{{\mathrm {d}}T}}{{{\mathrm {d}}X^ \ast }}$$

where *T* represents the set of transition parameters we estimated (colonization probability *γ* and persistence probability *φ*) calculated based on Eq. (1).

We used a global model to estimate species and study area-specific estimates of the relationship between our transition probabilities and the climate covariates. This model included our five climate covariates and their interactions with each of the species, regional, and site-level factors. We fit models with a Bayesian LASSO, to penalize over-fitting and minimize effects of multi-colinearity^66^. The LASSO penalty allowed us to include the range of factors we believed would affect climate sensitivity while controlling for model complexity and maximizing the model’s predictive power. Estimates from this model were used to generate estimates of sensitivity to changes in mean climate for each combination of species and study area in our data set.

We plotted sensitivity values across three different measures of space—geographic-space (latitude and longitude), climatic-space (local mean annual temperature and precipitation), and phylogenetic-space (on a phylogenetic tree). Mean annual temperatures and precipitation for locations were calculated using long-term 30-year average temperature and precipitation values for sites as determined using PRISM data. We used the same phylogeny that was used to impute missing life-history trait values in the previous section.

Finally, we assessed whether the climate sensitivities we estimated in combination with recent shifts in mean climate were sufficient to explain the overall declines in occupancy observed across species in this data set. We estimated the distribution of actual rates of decline using a hierarchical trend model^30,38^. We compared these to estimates of the expected climate-driven change in occurrence for the past 30 years estimated using two methods. First, we estimated the trend in mean annual values for each climate variables based on the linear regression of year vs. annual values for each of our climate variables. We combined the estimate of trend in mean climate (change in mean climate/year) with our measure of sensitivity (change in occupancy/change in mean climate) to calculate the expected change in occupancy per year.

8 $$\frac{{{\mathrm {d}}\psi }}{{{\mathrm {d}}t}} = \mathop {\sum }\limits_{ \in X^ \ast } \frac{{{\mathrm {d}}\psi }}{{{\mathrm {d}}X^ \ast }}\frac{{{\mathrm {d}}X^ \ast }}{{{\mathrm {d}}t}}$$

This quantifies the expected change in occurrence with a change in average climatic conditions.

Second, we estimated total climate effect by directly simulating annual occurrence values over the same 30-year period based on the estimated relationship between annual values of each of our climate variables and expected colonization and persistence probabilities from our global model results. We compared predicted trends based on climate change alone to the actual observed trends for each combination of species and study area in the dataset.

In addition to comparing overall average trend estimates, we wished to assess the degree to which variation in species-specific and location-specific trends were similar between the observed trends and the climate-driven predicted trends. To do this, we reran the observed trend model, using the predicted climate-driven trend as a predictor of actual trend. This allowed us to assess correlation, while accounting for uncertainty in the observed trend estimate.

### Testing assumptions

Finally, we examined how violations of assumptions affected sensitivity estimates using Monte Carlo-simulated data. A major goal of our study was to estimate sensitivity of occupancy probabilities to changes in climatic conditions. The measure of sensitivity we use is the expected rate of change in equilibrium occupancy probability given change in each of our five climatic variables. We calculated sensitivities using equations developed assuming that there is no random variation colonization and persistence probabilities (deterministic), and that mean colonization and persistence probabilities do not change outside of changes to the variable of interest (stationary). Approximating sensitivity assuming these two conditions is a typical approach used in many studies of ecological dynamics^39–41,67,68^. However, the assumption that deterministic and stationary sensitivity calculations are appropriate is not well tested for patch occupancy models of the type used here.

To better understand the assumptions and properties of our sensitivity estimators, we test how stochastic variation and non-stationary dynamics affect the sensitivity of occupancy to changes in underlying dynamics. For a range of scenarios, we estimate sensitivities using a numerical simulation approach^39^ comparing sensitivity for the deterministic and stationary case to alternatives in which either stochastic or non-stationary dynamics were included. As a baseline in each case we used all combinations of a set of reasonable colonization probabilities (0.018, 0.047, 0.119, 0.269, 0.378, and 0.5090) and persistence probabilities (0.500, 0.622, 0.731, 0.881, 0.953, and 0.982), for a total of 36 different scenarios.

First, we compared our baseline scenarios to one in which colonization and persistence were highly stochastic, varying from year-to-year. We simulated variation on a logit scale, assuming variation was randomly distributed with a standard deviation of 1. In the case of colonization or persistence equal to 0.5, this is the equivalent of 95% of the values coming between 0.12 and 0.88. This level of variability would only be expected to occur in our most stochastic data sets, such as the arroyo toad in California^17^. Thus it set an upper bound for potential bias. Results show that stochastic variation as sensitivities increase does lead to some underestimation of sensitivity as the magnitude of the sensitivity increases (Supplementary Fig. 4). The underestimation would not affect general conclusions and the region where underestimation occurs is well above the range where our values occur.

Second, we compared predictions of sensitivity of ending occupancy probability after 10 years for a times-series starting at equilibrium, and in which colonization and persistence were constant vs. where it changed systematically every year (i.e., non-stationary dynamics). For each of our 36 scenarios, we compared the baseline (constant colonization and persistence) vs. one in which either colonization or persistence increased or decreased by a value of 0.1 annually on a logit scale. This is the equivalent of the annual odds of colonization or persistence increasing to 2.72 times its starting value or decreasing to 1/2.72 times its original value. For this set of scenarios we see that on average the sensitivity is equivalent for stationary and non-stationary case, with some minor loss of precision (Supplementary Fig. 5). The overall correlation between the two measures is *r* > 0.99.

The simulations demonstrate that violations of the magnitude found in our study lead to small levels of bias and do not impact the conclusions made using these calculations (Supplementary Figs. 4 and 5). This suggests that our measure of sensitivity is a good predictor of how populations will respond under current conditions. However, as mean climate changes further, as other factors that affect local population dynamics change, and as new thresholds are met, sensitivity to change will also change. Thus, our results are best interpreted as measures of expected current rate of response to change.

### Code availability

The MCMC code (written in bugs language and run using JAGS) to run the main model is available in the supplementary files (Supplementary Data 1).

## Electronic supplementary material

Supplementary Information

Description of Additional Supplementary Files

Supplementary Dataset 1

Supplementary Dataset 2

Supplementary Dataset 3

## Data Availability

All data used in analysis available in public repositories or upon request. Climate data come from publicly available repositories maintained by other research groups (PRISM - http://prism.oregonstate.edu; Wang et al. 2009). Amphibian encounter data along with associated covariate data used in analysis is archived using Dryad (doi:10.5061/dryad.jt089hg). Location data for individual sites is not included because threatened and endangered species are included in the data set. However, information can be provided by directly contacting the authors. Species trait data can be found in the Supplementary Data 2
