# Supplementary material for: Quantifying climate sensitivity and climate-driven change in North American amphibian communities
Source: Nat Commun. 2018 Sep 25;9:3926. doi: 10.1038/s41467-018-06157-6 (PMC6156563; doi:10.1038/s41467-018-06157-6)
Supplement: Supplementary file 1 — Supplementary Information [file 41467_2018_6157_MOESM1_ESM.pdf]

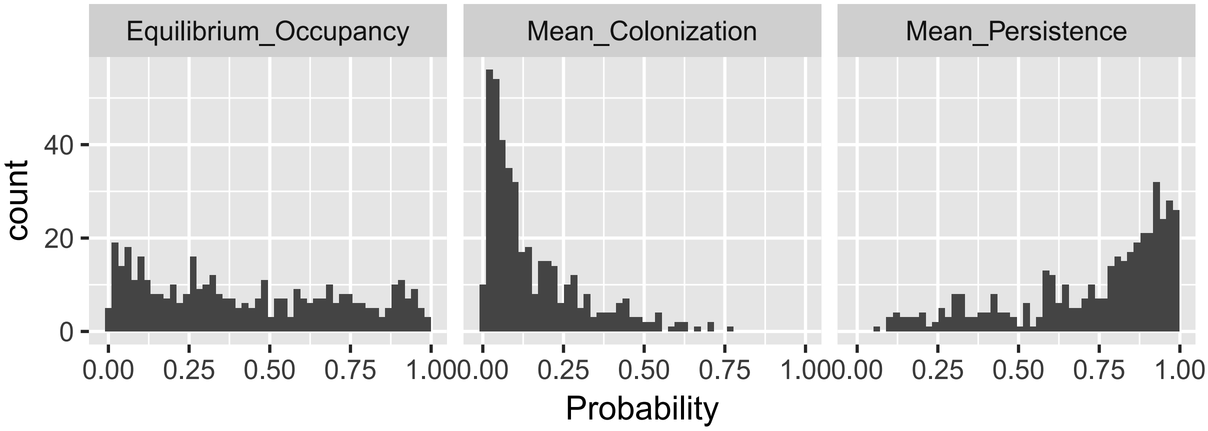

Supplementary Figure 1. **Estimated variation in dynamic occupancy model parameters** Histograms showing the range of estimated values for equilibrium occupancy, mean colonization, andmean persistence for each of the 407 time-series in the analysis. Each time-series represents a unique combination of study area and species.

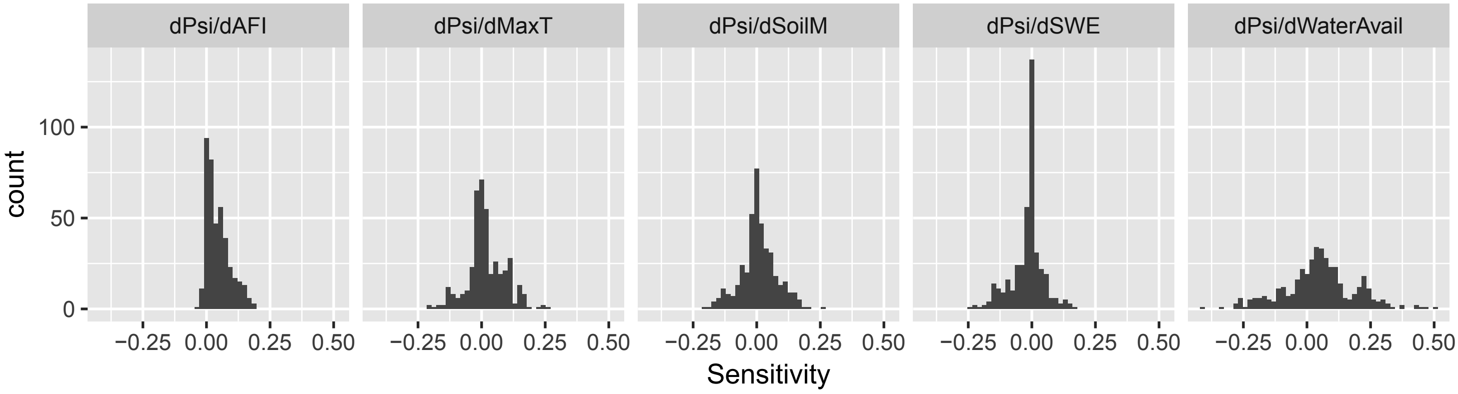

Supplementary Figure 2. **Estimated variation in sensitivity to climate change** Histograms showing the range of estimated values of sensitivity of equilibrium occupancy to each of the 5 climate variables for the 407 time-series in the analysis. Each time-series represents a unique combination of study area and species.

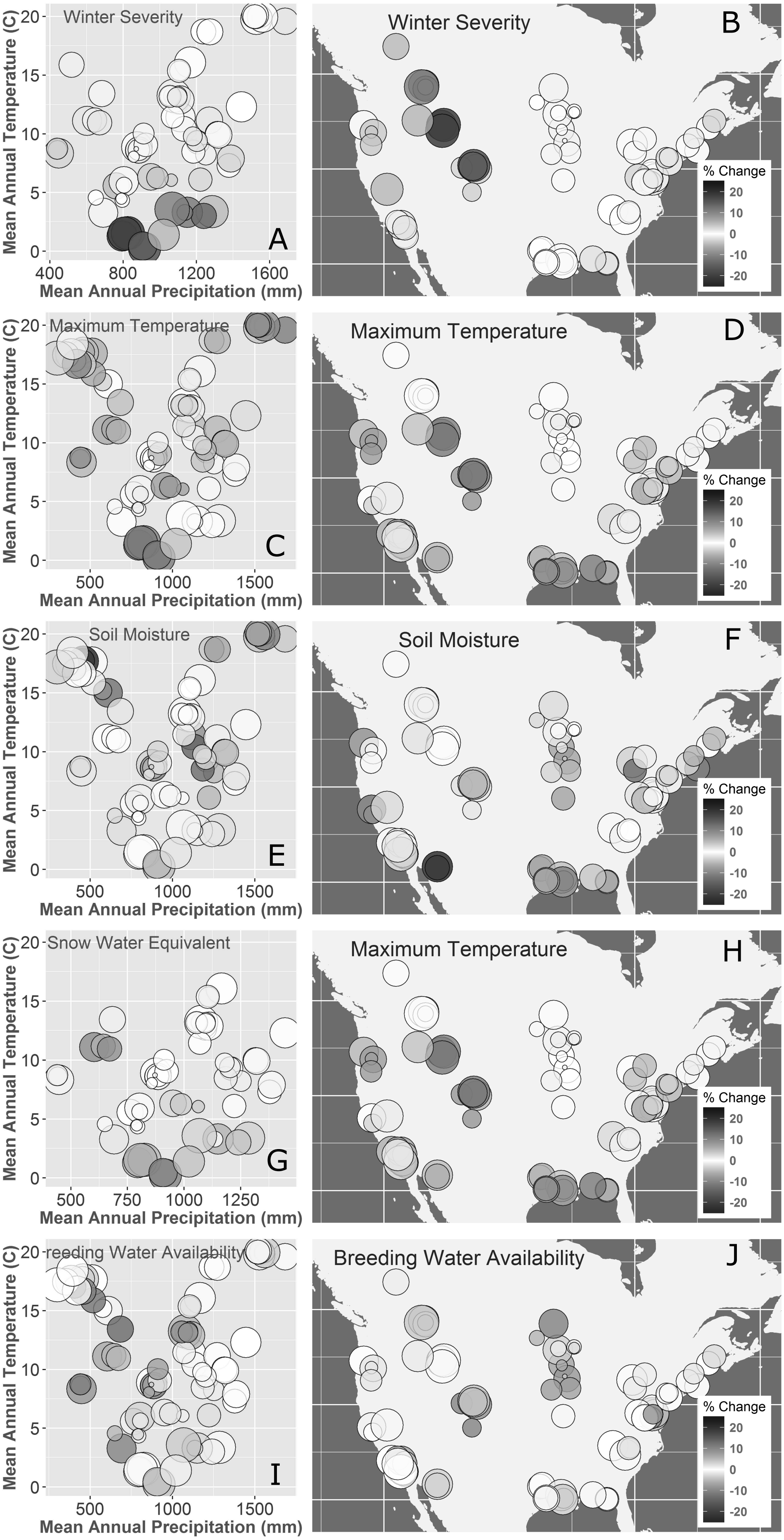

Supplementary Figure 3. **Predicted rates of change in species richness** Predicted 30-year change in species richness based on our dynamic species distribution model and observed changes in the mean for each of the climate variables considered separately from 1982 to 2012. Expected change if only the designated climate variable changes plotted in climate (A,C,E,G,I) and geographic space (B,D,F,H,J). Overall predicted change due to climate is shown in Fig. 5. Maps used in this figure were generated using R package `maps`.

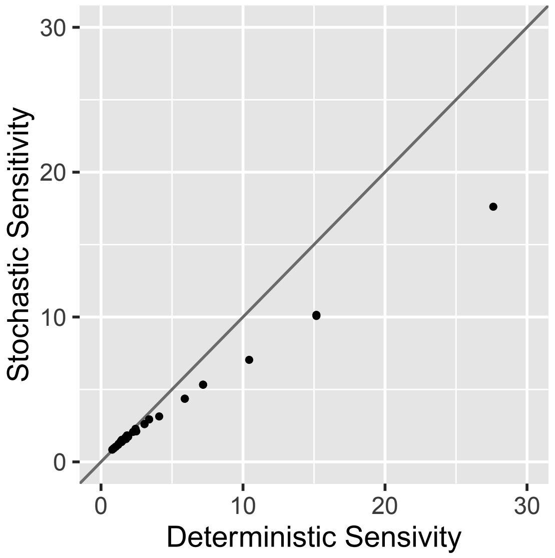

Supplementary Figures 4. **Effects of stochastic variation on sensitivity estimation** Comparison of estimated sensitivity assuming deterministic dynamics (i.e., colonization and persistence are constant) versus stochastic dynamics.

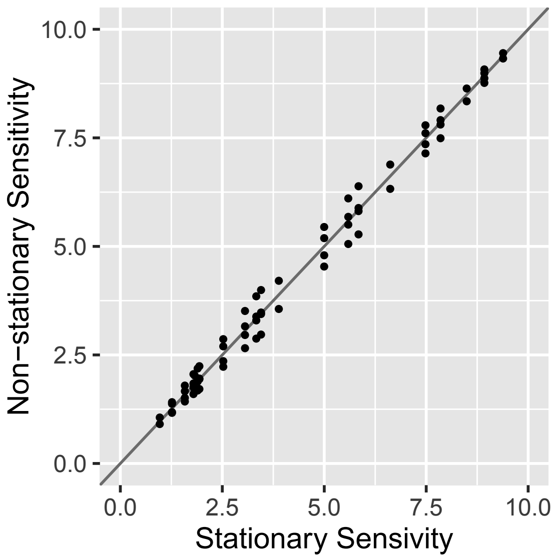

Supplementary Figures 5. **Effects of non-stationary dynamics on sensitivity estimation** Comparison of estimated sensitivity assuming stationary dynamics (i.e., mean colonization and persistence do not change through time) versus non-stationary dynamics.
