# Supplementary material for: Quantifying climate sensitivity and climate-driven change in North American amphibian communities
Source: Nat Commun. 2018 Sep 25;9:3926. doi: 10.1038/s41467-018-06157-6 (PMC6156563; doi:10.1038/s41467-018-06157-6)
Supplement: Supplementary file 2 — Description of Additional Supplementary Files [file 41467_2018_6157_MOESM2_ESM.pdf]

Description of Additional Supplementary Files:

Supplementary Data 1. **Code used to fit model in JAGS.** The code fits the full hierarchical model used to derive climate sensitivities.

Supplementary Data 2. **Species trait data.** A list of species included in analyses and trait values used in analyses.

Supplementary Data 3. **Time-series specific estimates.** Estimates of sensitivity and climate driven trend for each of the time-series in the analysis (i.e., a unique combination of species and study area.
